# Substrated inhomogeneous metasurfaces analysis using interaction constant method

**DOI:** 10.1038/s41598-023-28728-4

**Published:** 2023-01-28

**Authors:** Maryam Hesari-Shermeh, Bijan Abbasi-Arand

**Affiliations:** 1 Department of Electrical and Computer Engineering, Tarbiat Modares University, Tehran, 14115-194 Iran; 2 Iran National Science Foundation (INSF), Tehran, Iran

**Keywords:** Optics and photonics, Nanophotonics and plasmonics

## Abstract

Inhomogeneous metasurfaces as a periodic array of supercells in which each supercell consists of different types of particles are good candidates for increasing the bandwidth in many applications. However, the presence of a substrate is often apparent in many cases; therefore, analyzing substrated inhomogeneous metasurfaces is highly attractive and important. In this paper, an efficient analysis of the plane-wave scattering by inhomogeneous substrated metasurfaces is presented using interaction constant method (ICM). In our proposed method, we calculate the total effective polarizability tensors of inhomogeneous substrated metasurfaces using both the individual polarizabilities of each particle and the closed-form interaction coefficients that relate to the interactions of the particles with each other. Since the interaction constants are calculated analytically, this method is time effective for different arrangements of particles in supercells, and with different array periods. The reflectance and transmittance of different inhomogeneous metasurfaces have been obtained and compared to full-wave simulations by a commercial EM solver, here, and this has confirmed the accuracy of the numerical results of our proposed method. Moreover, in our last example, we present a wideband terahertz absorber, and analyze its structure with our method. It seems that our proposed method is a step forward in the analysis and design of inhomogeneous substrated metasurfaces, for various applications.

## Introduction

Metasurfaces have widespread applications in the fields of antenna and communication engineering^1–12,32,33^. In most applications, metasurfaces are fabricated on dielectric substrates to improve the structures’ mechanical robustness, and to make them more favorable for practical applications. Moreover, in many applications, to increase the bandwidth of the metasurfaces, multilayer structures or inhomogeneous metasurfaces consisting of different particles are used. Multilayer structures have many challenges, such as system complexity and reduced optimal-structure performance, while inhomogeneous metasurfaces, including particles with different arrangements in supercells, are alternative option for achieving optimal performance, and they provide additional resonances in their structures. Therefore, inhomogeneous metasurfaces are a good alternative to multilayer structures, with different profiles within each layer.

Indeed, using different particles with various arrangements inside supercells can cause additional resonances to occur in the frequency responses of the structures; therefore, analyzing substrated inhomogeneous metasurfaces, and understanding the behaviors of the electromagnetic waves in dealing with these structures, are important. There are various analytical and numerical methods presented in the literature for analyzing metasurfaces^13–17^; however, while the analytical methods are appropriate for designing and synthesizing metasurfaces, they are typically limited to simple configurations, and are not applicable for many engineered structures. Therefore, when increasing the complexities of the structures, numerical and semi-analytical methods must be applied^16–23^.

On the one hand, numerical methods can be used to solve different structures; however, these methods do not provide independent information about the inclusions themselves, or their arrangements. In contrast, a semi-analytical method has been presented for metasurface analysis based on an independent study of the electromagnetic behaviors of the constituting nanoparticles, and the interactions between them. Several references were using this approach for analyzing metasurfaces^18,20–24^. In^23^, this approach is used for analyzing inhomogeneous metasurfaces in free spaces; while in^21^, this method is extended to the case of periodic metasurfaces located on dielectric substrates, with normal incident plane waves. Yet, while inhomogeneous metasurfaces in the presence of substrates have many practical applications, the analysis of these metasurfaces has room for more effort.

In this paper, we extend the semi-analytical ICM to analyze inhomogeneous metasurfaces located at the interface between two dielectric media. When performing this method, the local effective polarizabilities of the supercells in the presence of a substrate should ultimately be calculated; and to do so, the individual polarizability tensors of each particle in the supercell, and the interaction coefficients of the particles inside the supercells, must be obtained. Hence, the main purpose of this paper is to calculate these coefficients in the presence of a dielectric substrate. Then, by using the local effective polarizabilities of the substrated supercell, and the interaction coefficients between the supercells, one can easily find the total effective polarizabilities of the substrated inhomogeneous metasurfaces. Consequently, it is possible to analyze the frequency response of an inhomogeneous metasurface, under normal illumination.

This paper is arranged as follows: in section “Theory”, the homogenization and characterization of inhomogeneous metasurfaces are explained, in the presence of substrates; and after presenting the analytical framework, in section “Results and discussion”, some general examples are provided that cover various cases of isotropic and bi-anisotropic metasurfaces. The results are then compared to the results of a full-wave simulator to confirm the accuracy of our proposed method. Finally, the paper is concluded in section “Conclusion”.

## Theory

The ICM presents an efficient homogenization model of metasurfaces^13^. In this method, a metasurface is considered as a homogenized surface with characteristic surface parameters, called the collective polarizability tensors. Therefore, an analysis of the electromagnetic response of a metasurface reduces to the calculation of these parameters; and by calculating the collective polarizability tensors of a metasurface, one can predict the reflection and transmission coefficients of the metasurface to external excitations.

A homogeneous metasurface is a structure in which the unit cell consists of an arbitrary particle that repeats with a specified period. In contrast, whenever referring to an inhomogeneous metasurface, in this paper, we mean a periodic array of supercells with different electrically small particles. Therefore, to model an inhomogeneous metasurface, we need to calculate the effective polarizabilities of one of the supercells, instead of the individual polarizabilities of the unit cell in the homogeneous structure.

**Figure 1:**
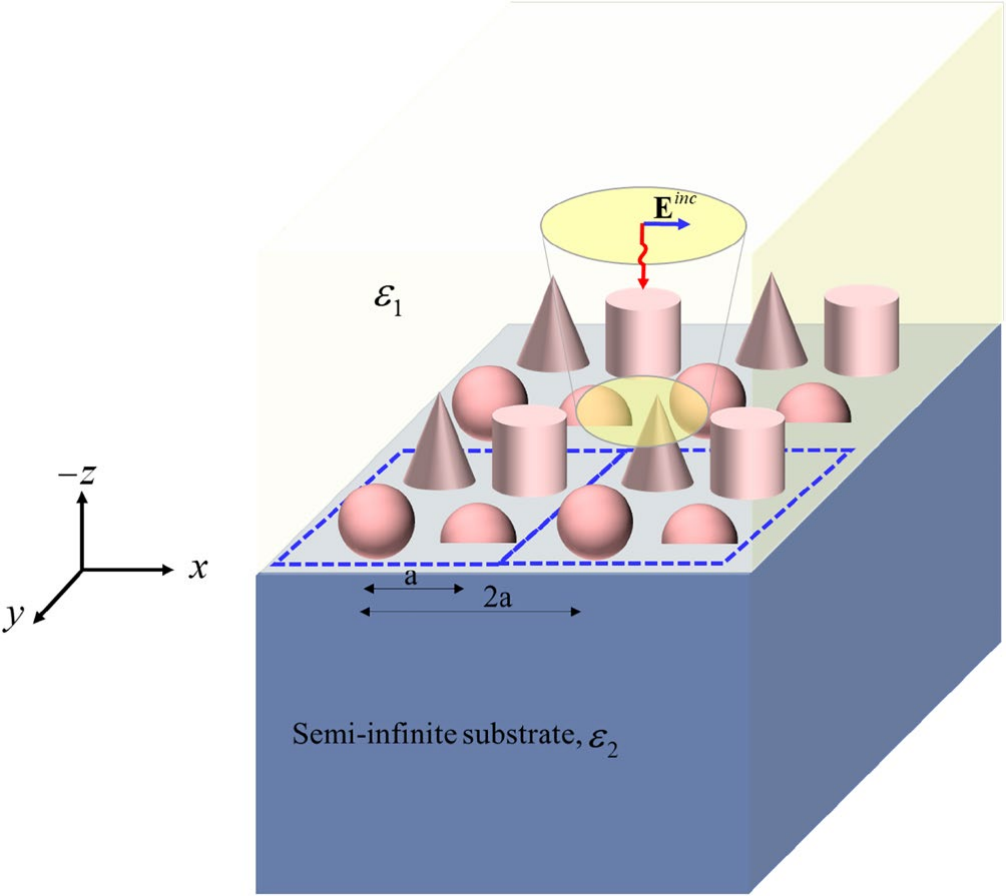
A general inhomogeneous metasurface sandwiched between two dielectric media.

Figure 1 represents an inhomogeneous metasurface located at the interface between two dielectric media, with permittivities,$\varepsilon_{1}$ and $\varepsilon_{2}$, under a normal incident plane wave; and as shown in the figure, each supercell is marked with a dashed line. Since different particles exist in the supercell, the incident fields induce different dipole moments on each particle; and as these induced dipole moments are calculated at the geometric center of each particle, the only significant moments are the electric and magnetic dipoles. Any other higher-order multipoles can be neglected^25,26^. Moreover, the dimensions of the supercells are small compared to the wavelength of the surrounding environment, so the local effective electric and magnetic moments of the supercell can be obtained by summing up the electric and magnetic moments of each particle in the supercell^29^. Therefore, according to the following equation, one can relate the effective dipole moments of each supercell to the local fields:

1 $$\left[ {\begin{array}{*{20}c} {{\mathbf{p}}_{{Sup}} } \\ {{\mathbf{m}}_{{Sup}} } \\ \end{array} } \right] = \left[ {\begin{array}{*{20}c} {\bar{\bar{\hat{\alpha }}}_{{loc}}^{{ee}} } & {\bar{\bar{\hat{\alpha }}}_{{loc}}^{{em}} } \\ {\bar{\bar{\hat{\alpha }}}_{{loc}}^{{me}} } & {\bar{\bar{\hat{\alpha }}}_{{loc}}^{{mm}} } \\ \end{array} } \right] \bullet \left[ {\begin{array}{*{20}c} {{\mathbf{E}}_{{}}^{{loc}} } \\ {{\mathbf{H}}_{{}}^{{loc}} } \\ \end{array} } \right].$$

where $\bar{\bar{\hat{\alpha }}}_{{loc}}$ is the local effective polarizability tensor of the supercell, and defined as follows:

2 $$\bar{\bar{\hat{\alpha }}}_{{loc}} = \left[ {\begin{array}{*{20}c} {\bar{\bar{\hat{\alpha }}}_{{loc}}^{{ee}} } & {\bar{\bar{\hat{\alpha }}}_{{loc}}^{{em}} } \\ {\bar{\bar{\hat{\alpha }}}_{{loc}}^{{me}} } & {\bar{\bar{\hat{\alpha }}}_{{loc}}^{{mm}} } \\ \end{array} } \right] = \left[ {\begin{array}{*{20}c} {\sum\limits_{{i = 1}}^{N} {\bar{\bar{\hat{\alpha }}}_{i}^{{ee}} } } & {\sum\limits_{{i = 1}}^{N} {\bar{\bar{\hat{\alpha }}}_{i}^{{em}} } } \\ {\sum\limits_{{i = 1}}^{N} {\bar{\bar{\hat{\alpha }}}_{i}^{{me}} } } & {\sum\limits_{{i = 1}}^{N} {\bar{\bar{\hat{\alpha }}}_{i}^{{mm}} } } \\ \end{array} } \right]$$

In Eq. (1), ${\mathbf{p}}_{Sup}$(${\mathbf{m}}_{Sup}$) and ${\mathbf{E}}_{{}}^{loc}$(${\mathbf{H}}_{{}}^{loc}$) are the electric (magnetic) moment of the supercell and the electric (magnetic) local field, respectively. Moreover, $\bar{\bar{\hat{\alpha }}}_{i}$ in Eq. (2) is the effective polarizability tensor of *i*th particle in the supercell, which is dependent on the individual polarizability tensors of the particle and the inter-supercells’ interaction coefficients (ISIC). In this paper, these parameters are calculated for an inhomogeneous metasurface in the presence of a substrate.

To calculate the local effective polarizability tensors of the supercell at the presence of a substrate, the individual polarizabilities of each particle should be calculated, first. There are different numerical and analytical methods available for calculating the polarizabilities of particles; however, analytical methods are usually complex and do not provide answers for an arbitrary particle. In contrast, numerical methods have lower speed and accuracy. Other potential methods are semi-analytical methods. In this paper, we present a semi-analytical method to calculate the polarizabilities of any particle that is located on a dielectric substrate.

Since the particles’ polarizabilities are their characteristic parameters, by knowing the polarizabilities, they can be applied as equivalent representations of the particles in any electromagnetic problem with plane-wave illuminations.

As mentioned previously, the polarizability tensors relate the electric (p) and magnetic (m) dipole moments to the local electromagnetic fields. So, in order to obtain the polarizabilities of a substrated particle, we need to calculate the local fields in the geometric center of the particle. Consequently, in this case, the local fields can be written as follows:

3 $$\begin{gathered} {\mathbf{\rm E}}_{loc}^{(p,s)} = {\mathbf{\rm E}}_{i}^{(p,s)} + {\mathbf{\rm E}}_{ref}^{(p,s)} \,\,, \hfill \\ {\mathbf{\rm H}}_{loc}^{(p,s)} = {\mathbf{\rm H}}_{i}^{(p,s)} + {\mathbf{\rm H}}_{ref}^{(p,s)} \,\,. \hfill \\ \end{gathered}$$

where ${\mathbf{\rm E}}_{i}^{(p,s)}$ and ${\mathbf{\rm E}}_{ref}^{(p,s)}$ are the incident and reflected electric fields from the substrate surface, respectively; and which are defined as follows for the (p) polarization:

4 $$\begin{gathered} {\mathbf{\rm E}}_{i}^{(p)} = {E}_{0} \left( {\cos \theta_{i} \begin{array}{*{20}c} {\hat{x} - } \\ \end{array} \sin \theta_{i} \begin{array}{*{20}c} {\hat{z}} \\ \end{array} } \right)\begin{array}{*{20}c} {e^{{ - j\begin{array}{*{20}c} {{\mathbf{k}}_{i} .{\mathbf{r}}} \\ \end{array} }} } \\ \end{array} , \hfill \\ {\mathbf{\rm E}}_{ref}^{(p)} = {E}_{0} {r}^{(p)} \left( {\cos \theta_{r} \begin{array}{*{20}c} {\hat{x}} \\ \end{array} + \sin \theta_{r} \begin{array}{*{20}c} {\hat{z}} \\ \end{array} } \right)\begin{array}{*{20}c} {e^{{ - j\begin{array}{*{20}c} {{\mathbf{k}}_{r} .{\mathbf{r}}} \\ \end{array} }} } \\ \end{array} , \hfill \\ \end{gathered}$$

In the above relations, ${r}^{(p)} = \frac{{\eta_{2} \cos \theta_{t} - \eta_{1} \cos \theta_{i} }}{{\eta_{2} \cos \theta_{t} + \eta_{1} \cos \theta_{i} }}e^{{ - 2jk_{1} \cos \theta_{i} z_{0} }}$ is the Fresnel reflection coefficient at the geometric center of the particle, while $\theta_{r}$ and $\theta_{t}$ are the angles of reflection and transmission, respectively. Similarly, the relations for the (s) polarization can also be written. By considering Eq. (1) for an individual particle and substituting Eq. (4) into the equation, the relations between the moments and the fields can be calculated, as follows:

5 $$\begin{gathered} p_{x} = \alpha_{xx}^{ee} \begin{array}{*{20}c} {{E}_{x} } \\ \end{array} + \alpha_{xz}^{ee} \begin{array}{*{20}c} {{E}_{z} } \\ \end{array} + \alpha_{xy}^{em} \begin{array}{*{20}c} {{\rm H}_{y} ,} \\ \end{array} \hfill \\ p_{y} = \alpha_{yx}^{ee} \begin{array}{*{20}c} {{E}_{x} } \\ \end{array} + \alpha_{yz}^{ee} \begin{array}{*{20}c} {{E}_{z} } \\ \end{array} + \alpha_{yy}^{em} \begin{array}{*{20}c} {{\rm H}_{y} } \\ \end{array} \hfill \\ p_{z} = \alpha_{zx}^{ee} \begin{array}{*{20}c} {{E}_{x} } \\ \end{array} + \alpha_{zz}^{ee} \begin{array}{*{20}c} {{E}_{z} } \\ \end{array} + \alpha_{zy}^{em} \begin{array}{*{20}c} {{\rm H}_{y} } \\ \end{array} , \hfill \\ m_{x} = \alpha_{xx}^{me} \begin{array}{*{20}c} {{E}_{x} } \\ \end{array} + \alpha_{xz}^{me} \begin{array}{*{20}c} {{E}_{z} } \\ \end{array} + \alpha_{xy}^{mm} \begin{array}{*{20}c} {{\rm H}_{y} } \\ \end{array} , \hfill \\ m_{y} = \alpha_{yx}^{me} \begin{array}{*{20}c} {{E}_{x} } \\ \end{array} + \alpha_{yz}^{me} \begin{array}{*{20}c} {{E}_{z} } \\ \end{array} + \alpha_{yy}^{mm} \begin{array}{*{20}c} {{\rm H}_{y} } \\ \end{array} , \hfill \\ m_{z} = \alpha_{zx}^{me} \begin{array}{*{20}c} {{E}_{x} } \\ \end{array} + \alpha_{zz}^{me} \begin{array}{*{20}c} {{E}_{z} } \\ \end{array} + \alpha_{zy}^{mm} \begin{array}{*{20}c} {{\rm H}_{y} } \\ \end{array} . \hfill \\ \end{gathered}$$

As seen in Eq. (5), 18 components of the particle's polarizability tensors can be calculated for plane-wave radiations with (p) polarizations. Similarly, if plane waves with (s) polarizations are radiated to the particle, the other polarizability components can be obtained.

Now, in order to calculate each of the components of the particle's polarizability tensors, according to Eq. (5), we need to radiate three plane-waves in three different directions because each of the relations in Eq. (5) has three unknown polarizabilities. To clarify the issue, we review the calculation process, wherein we first place the nanoparticle under a plane-wave radiation with (p) polarization in three directions, $\theta_{i,1}$, $\theta_{i,2}$ and $\theta_{i,3}$. Then, we calculate the electric and magnetic moments induced on the particle using the relations extracted in Supplementary Material (S1); and now, according to relation (5), we have:

6 $$p_{x,l} = \alpha_{xx}^{ee} \begin{array}{*{20}c} {{E}_{x,l} } \\ \end{array} + \alpha_{xz}^{ee} \begin{array}{*{20}c} {{E}_{z,l} } \\ \end{array} + \alpha_{xy}^{em} \begin{array}{*{20}c} {{\rm H}_{y,l} ,} \\ \end{array}$$

where l = 1, 2, 3 is related to the wave radiation in its corresponding directions. Next, by solving Eq. (6), each of the unknowns in this equation (i.e. $\alpha_{xx}^{ee}$, $\alpha_{xz}^{ee}$ and $\alpha_{xy}^{em}$) can be calculated; and other polarizability components can also be achieved in a similar way. We assume that $\theta_{i,1} = - \theta_{i,2} = 45^{ \circ }$ and $\theta_{i,3} = 0^{ \circ }$, and as a result, the polarizability components are obtained as follows:

7 $$\begin{gathered} \alpha_{qx}^{ee} = \frac{{2\left( {1 - r^{(p)} (\theta_{i,1} )} \right)p_{q,3} - \left( {1 - r^{(p)} (\theta_{i,3} )} \right)\left( {p_{q,1} + p_{i,2} } \right)}}{{{E}_{0} \left[ {2\left( {1 - r^{(p)} (\theta_{i,1} )} \right)\left( {1 + r^{(p)} (\theta_{i,3} )} \right) - \sqrt 2 \left( {1 + r^{(p)} (\theta_{i,1} )} \right)\left( {1 - r^{(p)} (\theta_{i,3} )} \right)} \right]}}, \hfill \\ \alpha_{qz}^{ee} = \frac{{p_{q,1} - p_{i,2} }}{{\sqrt 2 {E}_{0} \left( { - 1 + r^{p} (\theta_{i,1} )} \right)}}, \hfill \\ \alpha_{qy}^{em} = \frac{{\sqrt 2 \left( {1 + r^{(p)} (\theta_{i,1} )} \right)p_{q,3} - \left( {1 + r^{(p)} (\theta_{i,3} )} \right)\left( {p_{q,1} + p_{i,2} } \right)}}{{{E}_{0} \left[ {2\left( {1 - r^{(p)} (\theta_{i,1} )} \right)\left( {1 + r^{(p)} (\theta_{i,3} )} \right) - \sqrt 2 \left( {1 + r^{(p)} (\theta_{i,1} )} \right)\left( {1 - r^{(p)} (\theta_{i,3} )} \right)} \right]}}\eta_{1} , \hfill \\ \alpha_{qx}^{me} = \frac{{2\left( {1 - r^{(p)} (\theta_{i,1} )} \right)m_{q,3} - \left( {1 - r^{(p)} (\theta_{i,3} )} \right)\left( {m_{q,1} + m_{i,2} } \right)}}{{{E}_{0} \left[ {2\left( {1 - r^{(p)} (\theta_{i,1} )} \right)\left( {1 + r^{(p)} (\theta_{i,3} )} \right) - \sqrt 2 \left( {1 + r^{(p)} (\theta_{i,1} )} \right)\left( {1 - r^{(p)} (\theta_{i,3} )} \right)} \right]}}, \hfill \\ \alpha_{qz}^{me} = \frac{{m_{q,1} - m_{i,2} }}{{\sqrt 2 {E}_{0} \left( { - 1 + r^{(p)} (\theta_{i,1} )} \right)}}, \hfill \\ \alpha_{qy}^{mm} = \frac{{\sqrt 2 \left( {1 + r^{(p)} (\theta_{i,1} )} \right)m_{q,3} - \left( {1 + r^{(p)} (\theta_{i,3} )} \right)\left( {m_{q,1} + m_{i,2} } \right)}}{{{E}_{0} \left[ {2\left( {1 - r^{(p)} (\theta_{i,1} )} \right)\left( {1 + r^{(p)} (\theta_{i,3} )} \right) - \sqrt 2 \left( {1 + r^{(p)} (\theta_{i,1} )} \right)\left( {1 - r^{(p)} (\theta_{i,3} )} \right)} \right]}}\eta_{1} . \hfill \\ \end{gathered}$$

where q = x, y, z and $r^{(p)}$ is the Fresnel reflection coefficient. By calculating the individual polarizabilities for each particle in the supercell, in order to calculate the effective polarizabilities of the supercell, we need to calculate the ISIC.

**Figure 2:**
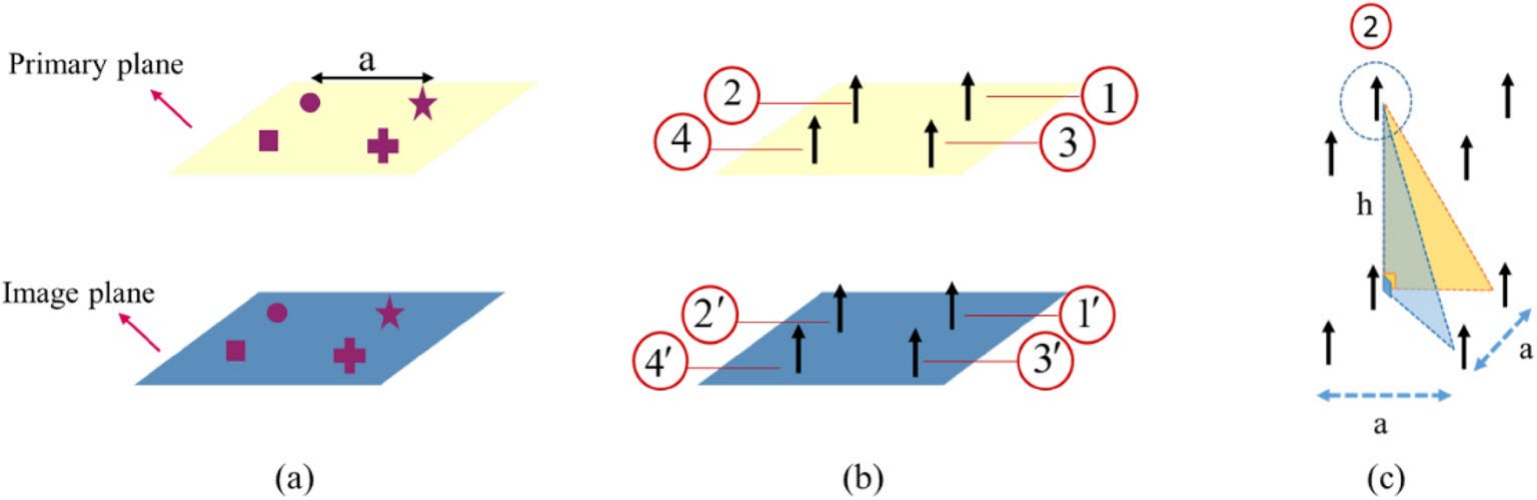
The selected supercell and its equivalent electric-dipole model.

Interaction coefficients calculate the effects of the other particles in the supercell at the location of each of the supercell’s particles. In the presence of a substrate, the effects of the substrate should also be considered in calculating these coefficients. To do so, according to Fig. 2, we use the image theorem to model the effects of the substrate. As can be seen in the figure, we have two planes: the primary plane, which contains all the particles inside the supercell that have been replaced by the equivalent electric and magnetic dipole moments; and the secondary plane or image plane, which represents the image of the primary dipoles.

On the other hand, when a particle is placed next to other particles, the local fields acting on each particle would be the superposition of the incident field and the interaction fields created by the other particles inside the supercell, at the location of that particle. The interaction fields, in general, for the *i*th particle in the supercell can be defined as follows:

8 $$\begin{gathered} {\mathbf{E}}_{i}^{{\text{int} }} = \bar{\bar{\beta }}_{S}^{{ee}} .\begin{array}{*{20}c} {\mathbf{p}} \\ \end{array} _{i} + \bar{\bar{\beta }}_{S}^{{em}} .\begin{array}{*{20}c} {\mathbf{m}} \\ \end{array} _{i} , \hfill \\ {\mathbf{H}}_{i}^{{\text{int} }} = \bar{\bar{\beta }}_{S}^{{me}} .\begin{array}{*{20}c} {\mathbf{p}} \\ \end{array} _{i} + \bar{\bar{\beta }}_{S}^{{mm}} .\begin{array}{*{20}c} {\mathbf{m}} \\ \end{array} _{i} \begin{array}{*{20}c} . \\ \end{array} \hfill \\ \end{gathered}$$

where $\bar{\bar{\beta }}_{S}^{{ee}}$, $\bar{\bar{\beta }}_{S}^{{em}}$, $\bar{\bar{\beta }}_{S}^{{me}}$ and $\bar{\bar{\beta }}_{S}^{{mm}}$ are the electric, magneto-electric, electromagnetic and magnetic supercell interaction coefficients, respectively. For metasurfaces placed on substrates, according to Fig. 2, the interaction fields inside the supercell include the interactions of the primary dipoles (the yellow sheet in Fig. 2) and the image dipoles (the blue sheet in Fig. 2), which can be written as follows:

9 $$\begin{gathered} {\mathbf{E}}_{i}^{int,primary} = \sum\limits_{\begin{subarray}{l} j = 1 \\ i \ne j \end{subarray} }^{N} {\left( {{\mathbf{E}}_{{}}^{{p_{j} }} + {\mathbf{E}}_{{}}^{{m_{j} }} } \right)} , \hfill \\ {\mathbf{E}}_{i}^{int,image} = \sum\limits_{\begin{subarray}{l} j = 1 \\ i \ne j \end{subarray} }^{N} {\left( {{\mathbf{E^{\prime}}}_{{}}^{{p_{j} }} + {\mathbf{E^{\prime}}}_{{}}^{{m_{j} }} } \right)} . \hfill \\ \end{gathered}$$

Similarly, we can write the equation for ${\mathbf{H}}_{i}^{int,primary}$ and ${\mathbf{H}}_{i}^{int,image}$, where ${\mathbf{E}}_{i}^{int,primary}$(${\mathbf{H}}_{i}^{int,primary}$) indicates the interaction electric (magnetic) field produced by the primary dipoles of the array, and ${\mathbf{E}}_{i}^{int,image}$(${\mathbf{H}}_{i}^{int,image}$) indicates the interaction electric (magnetic) field produced by the image dipoles — thereby representing the effects of the substrate. Moreover, ${\mathbf{E}}_{{}}^{{p_{j} }}$ and ${\mathbf{E}}_{{}}^{{m_{j} }}$ are the electric fields caused by the electric and magnetic dipoles, respectively.

As mentioned in the literature, the electromagnetic fields of an electric dipole are calculated as follows:

10 $$\begin{gathered} {\mathbf{E}}_{p} ({\mathbf{r}}) = \frac{1}{{4\pi \varepsilon_{0} }}\left\{ {k^{2} ({\mathbf{n}} \times {\mathbf{p}}) \times {\mathbf{n}}\frac{{e^{ - jkr} }}{r} + [3{\mathbf{n}}({\mathbf{n}}.{\mathbf{p}}) - {\mathbf{p}}](\frac{1}{{r^{3} }} + \frac{jk}{{r^{2} }})e^{ - jkr} } \right\}, \hfill \\ {\mathbf{H}}_{p} ({\mathbf{r}}) = - \frac{{k^{2} C_{0} }}{4\pi }\left\{ {({\mathbf{n}} \times {\mathbf{p}})(1 + \frac{1}{jkr})\frac{{e^{ - jkr} }}{r}} \right\}. \hfill \\ \end{gathered}$$

From the duality theorem, the electric and magnetic fields for the induced magnetic dipoles can easily be obtained. Then, by considering Eqs. (8) and (9), we can rewrite the electric and magnetic interaction fields as follows:

11 $$\begin{gathered} {\mathbf{E}}_{i}^{{int,primary}} = \sum\limits_{\begin{subarray}{l} j = 1 \\ j \ne i \end{subarray} }^{N} {\left( {\bar{\bar{\beta }}_{{0ji}}^{{ee}} \begin{array}{*{20}c} {{\mathbf{p}}_{j} } \\ \end{array} + \bar{\bar{\beta }}_{{0ji}}^{{em}} \begin{array}{*{20}c} {{\mathbf{m}}_{j} } \\ \end{array} } \right)} \hfill \\ {\mathbf{E}}_{i}^{{int,image}} = \sum\limits_{\begin{subarray}{l} j = 1 \\ i \ne j \end{subarray} }^{N} {\left( {\bar{\bar{\beta }}_{{ji}}^{{{\prime}ee}} \begin{array}{*{20}c} {{\mathbf{p}}_{j} } \\ \end{array} + \bar{\bar{\beta }}_{{ji}}^{{{\prime}em}} \begin{array}{*{20}c} {{\mathbf{m}}_{j} } \\ \end{array} } \right)} , \hfill \\ {\mathbf{H}}_{i}^{{int,primary}} = \sum\limits_{\begin{subarray}{l} j = 1 \\ j \ne i \end{subarray} }^{N} {\left( {\bar{\bar{\beta }}_{{0ji}}^{{me}} \begin{array}{*{20}c} {{\mathbf{p}}_{j} } \\ \end{array} + \bar{\bar{\beta }}_{{0ji}}^{{mm}} \begin{array}{*{20}c} {{\mathbf{m}}_{j} } \\ \end{array} } \right)} , \hfill \\ {\mathbf{H}}_{i}^{{int,image}} = \sum\limits_{\begin{subarray}{l} j = 1 \\ i \ne j \end{subarray} }^{N} {\left( {\bar{\bar{\beta }}_{{ji}}^{{{\prime}me}} \begin{array}{*{20}c} {{\mathbf{p}}_{j} } \\ \end{array} + \bar{\bar{\beta }}_{{ji}}^{{{\prime}mm}} \begin{array}{*{20}c} {{\mathbf{m}}_{j} } \\ \end{array} } \right)} , \hfill \\ \end{gathered}$$

where, $\bar{\bar{\beta }}_{{0ji}}^{{ee}}$, $\bar{\bar{\beta }}_{{0ji}}^{{em}}$, $\bar{\bar{\beta }}_{{0ji}}^{{me}}$ and $\bar{\bar{\beta }}_{{0ji}}^{{mm}}$ are the primary electric, magneto-electric, electro-magnetic and magnetic inter-supercell interaction coefficients for the *i*th particle, respectively. Moreover, $\bar{\bar{\beta }}_{{ji}}^{{{\prime}ee}}$, $\bar{\bar{\beta }}_{{ji}}^{{{\prime}em}}$, $\bar{\bar{\beta }}_{{ji}}^{{{\prime}me}}$ and $\bar{\bar{\beta }}_{{ji}}^{{{\prime}mm}}$ are the secondary electric, magneto-electric, electromagnetic, and magnetic inter-supercell interaction coefficient tensors, respectively.

The primary ISICs are calculated at the plane of the metasurface, where $${\mathbf{n}} = \begin{array}{*{20}c} {\cos \varphi \begin{array}{*{20}c} {\hat{x} + } \\ \end{array} } \\ \end{array} \begin{array}{*{20}c} {\sin \varphi \begin{array}{*{20}c} {\hat{y}} \\ \end{array} } \\ \end{array}$$, and may be expressed as:

12 $$\begin{gathered} \bar{\bar{\beta }}_{0}^{{ee}} = \left[ {\begin{array}{*{20}c} {\beta _{{0xx}}^{{ee}} } & {\beta _{{0xy}}^{{ee}} } & 0 \\ {\beta _{{0yx}}^{{ee}} } & {\beta _{{0yy}}^{{ee}} } & 0 \\ 0 & 0 & {\beta _{{0zz}}^{{ee}} } \\ \end{array} } \right],\begin{array}{*{20}c} {} \\ \end{array} \bar{\bar{\beta }}_{0}^{{me}} = \left[ {\begin{array}{*{20}c} 0 & 0 & {\beta _{{0xz}}^{{me}} } \\ 0 & 0 & {\beta _{{0yz}}^{{me}} } \\ {\beta _{{0zx}}^{{me}} } & {\beta _{{0zy}}^{{me}} } & 0 \\ \end{array} } \right], \hfill \\ \bar{\bar{\beta }}_{0}^{{mm}} = \frac{{\bar{\bar{\beta }}_{0}^{{ee}} }}{{\eta ^{2} }},\begin{array}{*{20}c} {} \\ \end{array} \bar{\bar{\beta }}_{0}^{{em}} = - \bar{\bar{\beta }}_{0}^{{me}} . \hfill \\ \beta _{{0xx,ji}}^{{ee}} = \left\{ {\left( {\frac{{jk}}{{r_{{ji}} }} + \frac{1}{{r_{{ji}}^{2} }}} \right)[3\cos ^{2} \varphi _{{ji}} - 1] + k^{2} \sin ^{2} \varphi _{{ji}} } \right\}\frac{{e^{{ - jkr_{{ji}} }} }}{{4\pi \varepsilon _{0} r_{{ji}} }}\,, \hfill \\ \beta _{{0yy,ji}}^{{ee}} = \left\{ {\left( {\frac{{jk}}{{r_{{ji}} }} + \frac{1}{{r_{{ji}}^{2} }}} \right)[3\sin ^{2} \varphi _{{ji}} - 1] + k^{2} \cos ^{2} \varphi _{{ji}} } \right\}\frac{{e^{{ - jkr_{{ji}} }} }}{{4\pi \varepsilon _{0} r_{{ji}} }}\,, \hfill \\ \beta _{{0xy,ji}}^{{ee}} = \beta _{{0yx,ji}}^{{ee}} = \left\{ {\left( {\frac{{jk}}{{r_{{ji}} }} + \frac{1}{{r_{{ji}}^{2} }}} \right)3\sin \varphi _{{ji}} \cos \varphi _{{ji}} + k^{2} \sin \varphi _{{ji}} \cos \varphi _{{ji}} } \right\}\frac{{e^{{ - jkr_{{ji}} }} }}{{4\pi \varepsilon _{0} r_{{ji}} }}\,, \hfill \\ \beta _{{0zz,ji}}^{{ee}} = \left\{ { - \left( {\frac{{jk}}{{r_{{ji}} }} + \frac{1}{{r_{{ji}}^{2} }}} \right) + k^{2} } \right\}\frac{{e^{{ - jkr_{{ji}} }} }}{{4\pi \varepsilon _{0} r_{{ji}} }}\,, \hfill \\ \beta _{{0xz,ji}}^{{me}} = - \beta _{{zx,ji}}^{{me}} = - j\omega \left( {jk + \frac{1}{{r_{{ji}} }}} \right)\sin \varphi \frac{{e^{{ - jkr_{{ji}} }} }}{{4\pi \varepsilon _{0} r_{{ji}} }}\,, \hfill \\ \beta _{{0yz,ji}}^{{me}} = - \beta _{{0zy,ji}}^{{me}} = j\omega \left( {jk + \frac{1}{{r_{{ji}} }}} \right)\cos \varphi \frac{{e^{{ - jkr_{{ji}} }} }}{{4\pi \varepsilon _{0} r_{{ji}} }}\,, \hfill \\ \end{gathered}$$

where $r_{ji}$ and $\varphi_{ji}$ are the distances and relative angles between the *j*th and *i*th primary dipoles, respectively. Moreover, it is worth noting that the electromagnetic and magneto-electric interaction coefficients would be non-zero due to the different types of particles in the supercell. In Eq. (12), according to the positions of each particle, the variables, $r_{ji}$ and $\varphi_{ji}$, could be determined.

For example, when considering a supercell with *N* particles, *N-*1 interaction fields would be determined for each particle; so, we would have $N \times (N - 1)$ interaction coefficients. Therefore, to determine the interaction coefficients of each of the supercell's particles according to their Cartesian coordinates, using Eq. (13), the values of $r_{ji}$ and $\varphi_{ji}$ for each particle can be obtained. By substituting these values into Eq. (12), the primary ISICs can be calculated.

13 $$\begin{gathered} \varphi_{ij} = 180^{ \circ } + \varphi_{ji} ,\begin{array}{*{20}c} {} & {j > i} \\ \end{array} \hfill \\ r_{ij} = r_{ji} . \hfill \\ \end{gathered}$$

However, for calculating the secondary ISICs, we have $${\mathbf{n}} = \sin \theta \begin{array}{*{20}c} {\cos \varphi \begin{array}{*{20}c} {\hat{x} + } \\ \end{array} } \\ \end{array} \sin \theta \begin{array}{*{20}c} {\sin \varphi \begin{array}{*{20}c} {\hat{y} + \cos \theta \begin{array}{*{20}c} {\hat{z}} \\ \end{array} } \\ \end{array} } \\ \end{array}$$. By substituting the general form of **n** in Eq. (10) and considering that the image dipole moments have a magnitude of $\frac{{\varepsilon_{2} - \varepsilon_{1} }}{{\varepsilon_{2} + \varepsilon_{1} }}$- times the primary dipole and depending on the direction of the primary dipole, the image dipole could be in phase or out of phase, and we have:

14 $$\begin{gathered} {\beta^{\prime}}_{xx,ji}^{ee} = \frac{{ - C_{i} e^{{ - jk{r^{\prime}}_{ji} }} }}{{4\pi \varepsilon_{0} {r^{\prime}}_{ji} }}\left\{ {\left( {\frac{1}{{{r^{\prime}}_{ji}^{2} }} + \frac{jk}{{{r^{\prime}}_{ji} }}} \right)\left( {3\sin^{2} {\theta^{\prime}}_{ji} \begin{array}{*{20}c} {\cos^{2} {\varphi^{\prime}}_{ji} - 1} \\ \end{array} } \right) + k^{2} \left( {\sin^{2} {\theta^{\prime}}_{ji} \begin{array}{*{20}c} {\sin^{2} {\varphi^{\prime}}_{ji} } \\ \end{array} + \cos^{2} {\theta^{\prime}}_{ji} } \right)} \right\}, \hfill \\ {\beta^{\prime}}_{xy,ji}^{ee} = \frac{{ - C_{i} e^{{ - jk{r^{\prime}}_{ji} }} }}{{4\pi \varepsilon_{0} {r^{\prime}}_{ji} }}\left\{ {3\left( {\frac{1}{{{r^{\prime}}_{ji}^{2} }} + \frac{jk}{{{r^{\prime}}_{ji} }}} \right)\sin^{2} {\theta^{\prime}}_{ji} \sin {\varphi^{\prime}}_{ji} \cos {\varphi^{\prime}}_{ji} - k^{2} \sin^{2} {\theta^{\prime}}_{ji} \begin{array}{*{20}c} {\sin {\varphi^{\prime}}_{ji} } \\ \end{array} \begin{array}{*{20}c} {\cos {\varphi^{\prime}}_{ji} } \\ \end{array} } \right\}, \hfill \\ {\beta^{\prime}}_{xz,ji}^{ee} = \frac{{C_{i} e^{{ - jk{r^{\prime}}_{ji} }} }}{{4\pi \varepsilon_{0} {r^{\prime}}_{ji} }}\left\{ {3\left( {\frac{1}{{{r^{\prime}}_{ji}^{2} }} + \frac{jk}{{{r^{\prime}}_{ji} }}} \right)\sin {\theta^{\prime}}_{ji} \begin{array}{*{20}c} {\cos {\theta^{\prime}}_{ji} } \\ \end{array} \cos {\varphi^{\prime}}_{ji} - k^{2} \sin {\theta^{\prime}}_{ji} \begin{array}{*{20}c} {\cos {\theta^{\prime}}_{ji} } \\ \end{array} \begin{array}{*{20}c} {\cos {\varphi^{\prime}}_{ji} } \\ \end{array} } \right\}, \hfill \\ {\beta^{\prime}}_{yy,ji}^{ee} = \frac{{ - C_{i} e^{{ - jk{r^{\prime}}_{ji} }} }}{{4\pi \varepsilon_{0} {r^{\prime}}_{ji} }}\left\{ {\left( {\frac{1}{{{r^{\prime}}_{ji}^{2} }} + \frac{jk}{{{r^{\prime}}_{ji} }}} \right)\left( {3\sin^{2} {\theta^{\prime}}_{ji} \begin{array}{*{20}c} {\sin^{2} {\varphi^{\prime}}_{ji} - 1} \\ \end{array} } \right) + k^{2} \left( {\sin^{2} {\theta^{\prime}}_{ji} \begin{array}{*{20}c} {\cos^{2} {\varphi^{\prime}}_{ji} } \\ \end{array} + \cos^{2} {\theta^{\prime}}_{ji} } \right)} \right\}, \hfill \\ {\beta^{\prime}}_{yz,ji}^{ee} = \frac{{C_{i} e^{{ - jk{r^{\prime}}_{ji} }} }}{{4\pi \varepsilon_{0} {r^{\prime}}_{ji} }}\left\{ {3\left( {\frac{1}{{{r^{\prime}}_{ji}^{2} }} + \frac{jk}{{{r^{\prime}}_{ji} }}} \right)\sin {\theta^{\prime}}_{ji} \begin{array}{*{20}c} {\cos {\theta^{\prime}}_{ji} } \\ \end{array} \sin {\varphi^{\prime}}_{ji} - k^{2} \sin {\theta^{\prime}}_{ji} \begin{array}{*{20}c} {\cos {\theta^{\prime}}_{ji} } \\ \end{array} \begin{array}{*{20}c} {\sin {\varphi^{\prime}}_{ji} } \\ \end{array} } \right\}, \hfill \\ {\beta^{\prime}}_{zz,ji}^{ee} = \frac{{C_{i} e^{{ - jk{r^{\prime}}_{ji} }} }}{{4\pi \varepsilon_{0} {r^{\prime}}_{ji} }}\left\{ {\left( {\frac{1}{{{r^{\prime}}_{ji}^{2} }} + \frac{jk}{{{r^{\prime}}_{ji} }}} \right)\left( {3\cos^{2} {\theta^{\prime}}_{ji} \begin{array}{*{20}c} { - 1} \\ \end{array} } \right) + k^{2} \sin^{2} {\theta^{\prime}}_{ji} } \right\}, \hfill \\ {\beta^{\prime}}_{yx,ji}^{ee} = {\beta^{\prime}}_{xy,ji}^{ee} ,\begin{array}{*{20}c} {{\beta^{\prime}}_{zx,ji}^{ee} = - {\beta^{\prime}}_{xz,ji}^{ee} ,} \\ \end{array} \begin{array}{*{20}c} {{\beta^{\prime}}_{zy,ji}^{ee} = - {\beta^{\prime}}_{yz,ji}^{ee} ,} \\ \end{array} \hfill \\ {\beta^{\prime}}_{xy,ji}^{me} = - {\beta^{\prime}}_{yx,ji}^{me} = - j\omega C_{i} (jk + \frac{1}{{{r^{\prime}}_{ji} }})\cos {\theta^{\prime}}_{ji} \frac{{e^{{ - jk{r^{\prime}}_{ji} }} }}{{4\pi {r^{\prime}}_{ji} }}, \hfill \\ {\beta^{\prime}}_{xz,ji}^{me} = {\beta^{\prime}}_{zx,ji}^{me} = - j\omega C_{i} (jk + \frac{1}{{{r^{\prime}}_{ji} }})\sin {\theta^{\prime}}_{ji} \begin{array}{*{20}c} {\sin {\varphi^{\prime}}_{ji} } \\ \end{array} \frac{{e^{{ - jk{r^{\prime}}_{ji} }} }}{{4\pi {r^{\prime}}_{ji} }}, \hfill \\ {\beta^{\prime}}_{yz,ji}^{me} = {\beta^{\prime}}_{zy,ji}^{me} = j\omega C_{i} (jk + \frac{1}{{{r^{\prime}}_{ji} }})\sin {\theta^{\prime}}_{ji} \begin{array}{*{20}c} {\cos {\varphi^{\prime}}_{ji} } \\ \end{array} \frac{{e^{{ - jk{r^{\prime}}_{ji} }} }}{{4\pi {r^{\prime}}_{ji} }}, \hfill \\ \end{gathered}$$

where $r^{\prime}_{ji}$, $\theta^{\prime}_{ji}$, and $\varphi^{\prime}_{ji}$ are the distances and relative angles between the images of *j*th dipole and *i*th primary dipole, respectively. Moreover, $C_{i}$ is the contrast factor, which is equal to $\frac{{\varepsilon_{2} - \varepsilon_{1} }}{{\varepsilon_{2} + \varepsilon_{1} }}$. To better understand the notation, here, the interaction coefficients on particle number 2, in a supercell with 4 different particles are labeled as: $\beta^{\prime}_{12}$,$\beta^{\prime}_{32}$ and $\beta^{\prime}_{42}$, which indicate the interactions of the images of the dipole numbers 1, 3 , and 4 on the primary dipole number 2, respectively. See Fig. 2c for more clarifications.

Finally, by summing up the primary and secondary interaction coefficients, we can calculate the ISICs in the presence of the substrate with the following:

15 $$\begin{gathered} \bar{\bar{\beta }}_{S}^{{ee}} = \left[ {\begin{array}{*{20}c} {\beta _{{xx}}^{{ee}} } & {\beta _{{xy}}^{{ee}} } & {\beta_{{xz}}^{{{\prime}ee}} } \\ {\beta _{{yx}}^{{ee}} } & {\beta _{{yy}}^{{ee}} } & {\beta_{{yz}}^{{{\prime}ee}} } \\ {\beta_{{zx}}^{{{\prime}ee}} } & {\beta_{{zy}}^{{{\prime}ee}} } & {\beta _{{zz}}^{{ee}} } \\ \end{array} } \right],\bar{\bar{\beta }}_{S}^{{me}} = \left[ {\begin{array}{*{20}c} 0 & {\beta_{{xy}}^{{{\prime}me}} } & {\beta _{{xz}}^{{me}} } \\ {\beta_{{yx}}^{{{\prime}me}} } & 0 & {\beta _{{yz}}^{{me}} } \\ {\beta _{{zx}}^{{me}} } & {\beta _{{zy}}^{{me}} } & 0 \\ \end{array} } \right], \hfill \\ \bar{\bar{\beta }}_{S}^{{mm}} = \frac{{\bar{\bar{\beta }}_{S}^{{ee}} }}{{\eta ^{2} }},\begin{array}{*{20}c} {} \\ \end{array} \bar{\bar{\beta }}_{S}^{{em}} = \bar{\bar{\beta }}_{S}^{{me}} , \hfill \\ \end{gathered}$$

where $$\beta_{mn}^{pq} = \beta_{0mn}^{pq} + {\beta^{\prime}{_{mn}^{pq}}}$$, $$m = x,y,z$$, $$n = x,y,z$$,$$p = e,m$$, and $$q = e,m$$.

Notice that, for the case of an inhomogeneous metasurface in the presence of a substrate (whose interaction coefficients are extracted in this paper), it can be observed that most of the interaction tensors are non-zero. On the other hand, most of the tangential and normal dipoles are seen to interact with each other.

After calculating the individual polarizability tensors of each particle in the supercell, and the ISICs in the presence of a substrate, one can use the procedure that we obtained and presented in Supplementary Material (S2) to calculate the local effective polarizabilities of each particle in the supercell, at the presence of a substrate. Then, to calculate the total effective polarizabilities of the substrated inhomogeneous metasurface using ICM, one can easily use the local effective polarizabilities of the substrated supercell (which is calculated in this paper) and the conventional interaction constants at the presence of a substrate that we presented in^21^. In fact, the array can be considered as a conventional array of supercells with a new period,*a'*, where this new period is the center-to-center distance between any two adjacent supercells. As it was mentioned in^18,21^, for any arbitrary nanoparticle in a defined frequency range, as long as the higher-order multipoles are not excited or can be negligible, *h/a'* < 0.75 and *ka*' < 3, the results of the proposed method are accurate, where *h* and *k* are the distance between the primary dipole and its image, and wave number, respectively. In fact, considering the conditions mentioned for this method in the current paper, this method no longer has a limit on frequency. Finally, by calculating the total effective polarizabilities of the inhomogeneous metasurface, one can obtain the reflection and transmission coefficients using the equation mentioned in^18^, for normal illuminations.

## Results and discussion

In the previous section, we showed how to calculate the local effective polarizabilities of a supercell, and also how to analyze the behavior of an inhomogeneous metasurface in the presence of a substrate under a normal plane wave, using our proposed ICM. Inhomogeneity, in supercells, can have various causes; whereby, using particles with different geometries, or using specific particles with different dimensions, or using the same particles with different constituent materials, or using non-square layouts in the supercells, can each cause inhomogeneities in the outcomes of the final supercells.

In this section, first, two inhomogeneous metasurfaces are studied to verify the efficiency and accuracy of our proposed method; and the results of our proposed method are compared to the results of a full-wave numerical simulator. Then a broadband absorber using an inhomogeneous substrated supercell is presented to provide a practical example of inhomogeneous metasurfaces. It is worth mentioning that our proposed approach is general, and can be used for metasurfaces with arbitrary particles that satisfy the conditions noted in the previous section. These examples are therefore only provided to validate the proposed approach.

### An array of silicon- and gold-spherical nanoparticles

**Figure 3:**
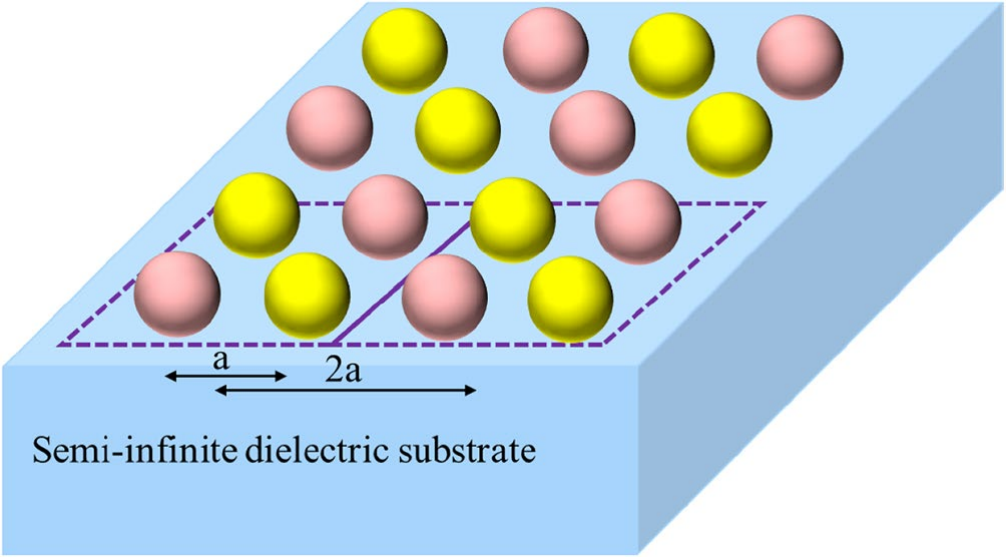
A substrated inhomogeneous metasurface consisting of combined silicon and gold nanoparticles with radii of 40 nm.

In the first example, consider an inhomogeneous substrated metasurface including a combined array of silicon- and gold-spherical nanoparticles, located on an infinite SiO2 substrate, as shown in Fig. 3. According to the figure, the center-to-center distance, *a*, between any adjacent particles is 150 nm, and the permittivities of these noted materials have been taken from^30,31^. In^12^, we discussed the effects of the materials on the polarizability tensors of the particles; while as mentioned in^12^, the main polarizabilities of the plasmonic nanoparticles with symmetrical shapes, such as nano-spheres or nano-cylinders with dimensions considered in this paper, are the electric polarizabilities. In contrast, high-index dielectric nanoparticles include both electric and magnetic polarizabilities.

**Figure 4:**
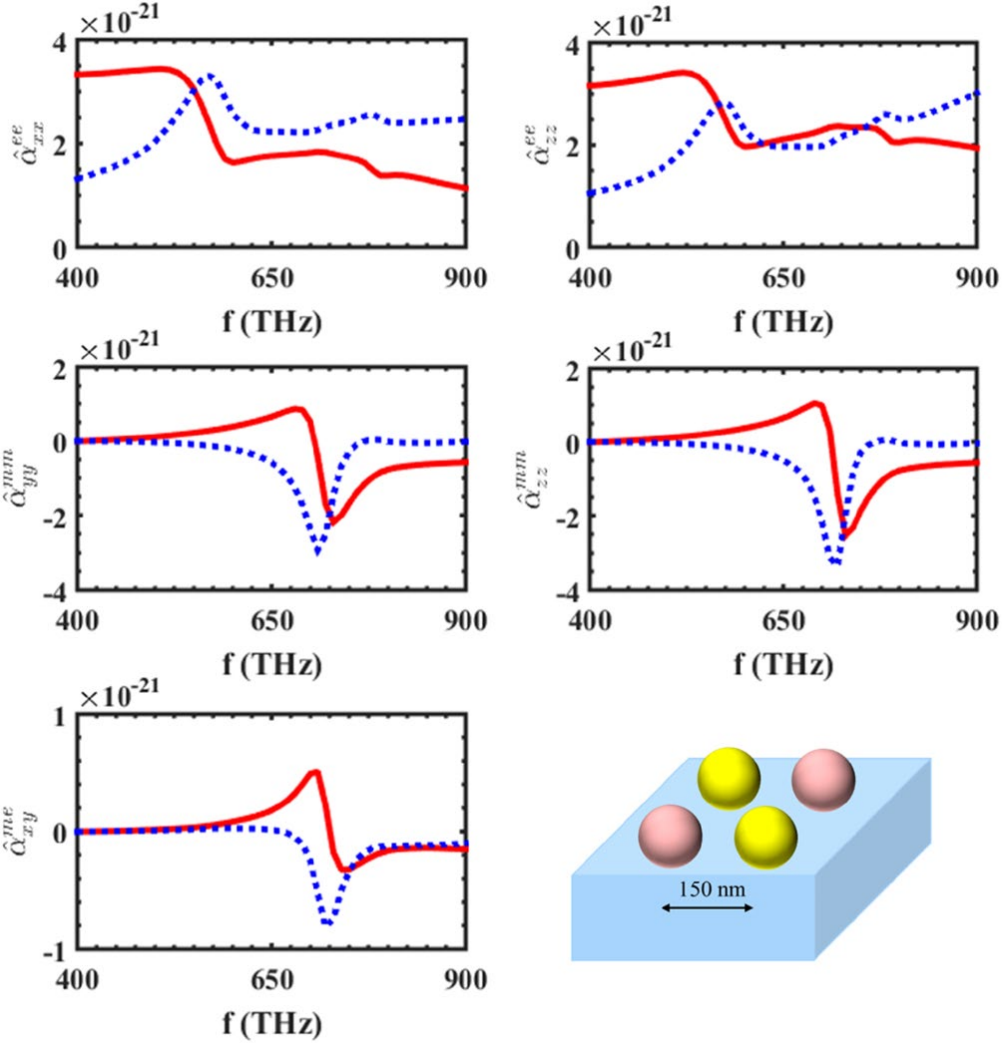
The major polarizabilities of the supercell shown in Fig. 3 placed on an infinite substrate, with $\varepsilon_{r} = 2.25$**.** The center-to-center distance of the elements is considered to be 150 nm.

**Figure 5:**
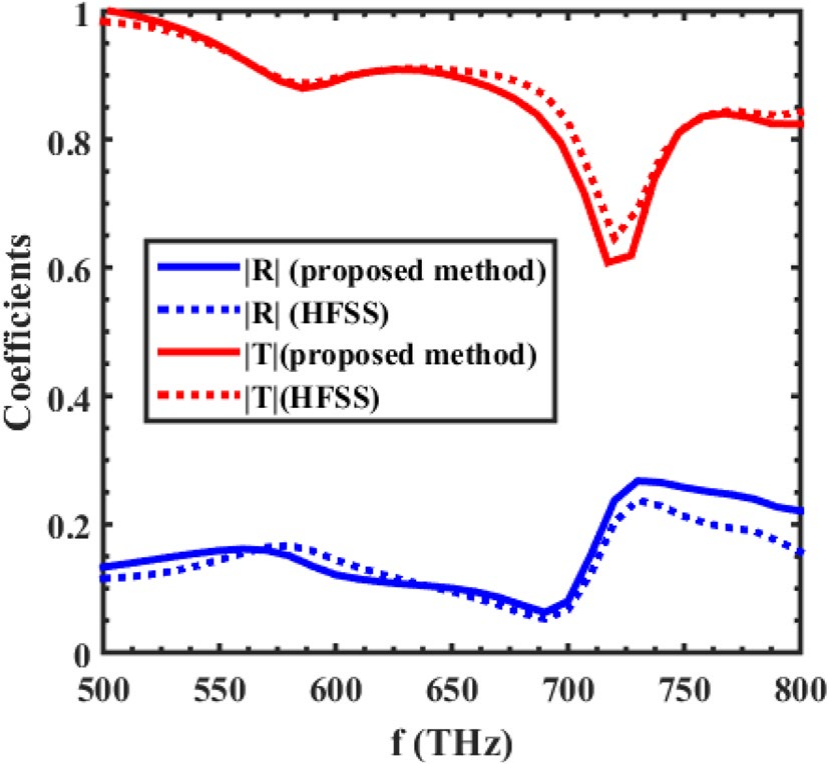
The reflection and transmission coefficients of the supercell shown in Fig. 3. The center-to-center distance of the elements is considered to be 150 nm.

To analyze this substrated inhomogeneous metasurface using our proposed method, we first need to calculate the individual polarizability tensors of the supercell's particles. Then, by calculating the ISICs, in the presence of a substrate, the local effective polarizabilities of the supercell can be calculated. After that, the characteristic parameters of the metasurface can be obtained by calculating the conventional interaction constants. Figure 4 shows the local effective polarizability of the supercell mentioned in Fig. 3, and as seen in the figure, the electric polarizability has two resonances, while the magnetic polarizability only has one resonance. Since both the gold and silicon spheres have electrical resonances, and their resonance frequencies are different, it is to be expected that the two resonances would be observed in the electrical polarizability of the supercell. Moreover, as mentioned previously, for this dimension of spheres, only the silicon spheres would have a dominant magnetic resonance; so, the magnetic polarizability of the supercell would only have one magnetic resonance. The electromagnetic polarizability would also be excited due to the presence of the substrate (through the bianisotropic effect caused by the substrate), and the existence of inhomogeneity in the supercell. The reflection and transmission coefficients of this array are shown in Fig. 5, and as the figure shows, our proposed method’s results are in good agreement with the results of a full-wave numerical simulator, HFSS.

### An array of gold spherical and single-split nano-cone particles

**Figure 6:**
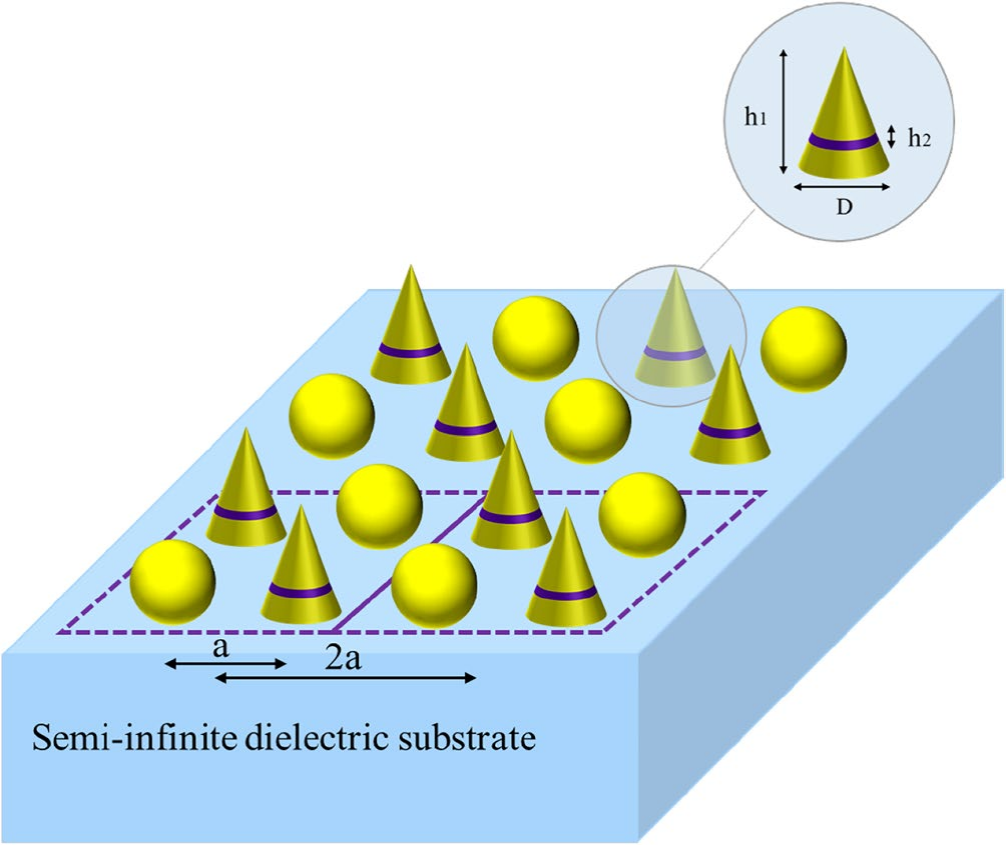
A substrated inhomogeneous metasurface consisting of gold spherical and single-split cone particles. The single- split nanocone, with $$D = 60\begin{array}{*{20}c} {nm,\begin{array}{*{20}c} {h_{1} = 100\begin{array}{*{20}c} {nm,\begin{array}{*{20}c} {h_{2} = 20\begin{array}{*{20}c} {nm,} \\ \end{array} } \\ \end{array} } \\ \end{array} } \\ \end{array} } \\ \end{array}$$ and the spherical particles with radii of 30 nm.

**Figure 7:**
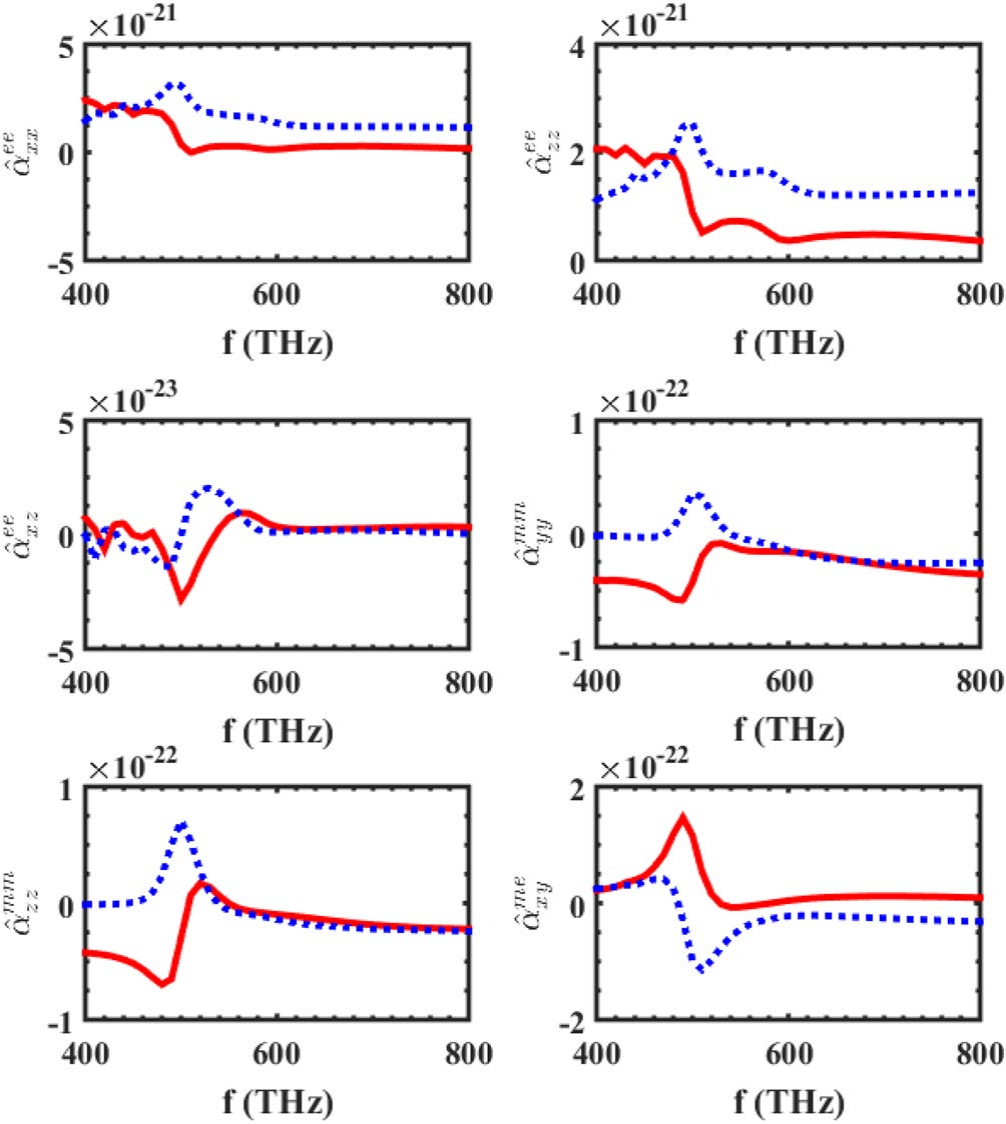
The main component of the polarizabilities of the supercell shown in Fig. 6 placed on an infinite SiO_2_ substrate, with $\varepsilon_{r} = 2.25$**.** The center-to-center distance of the elements is considered to be 150 nm.

**Figure 8:**
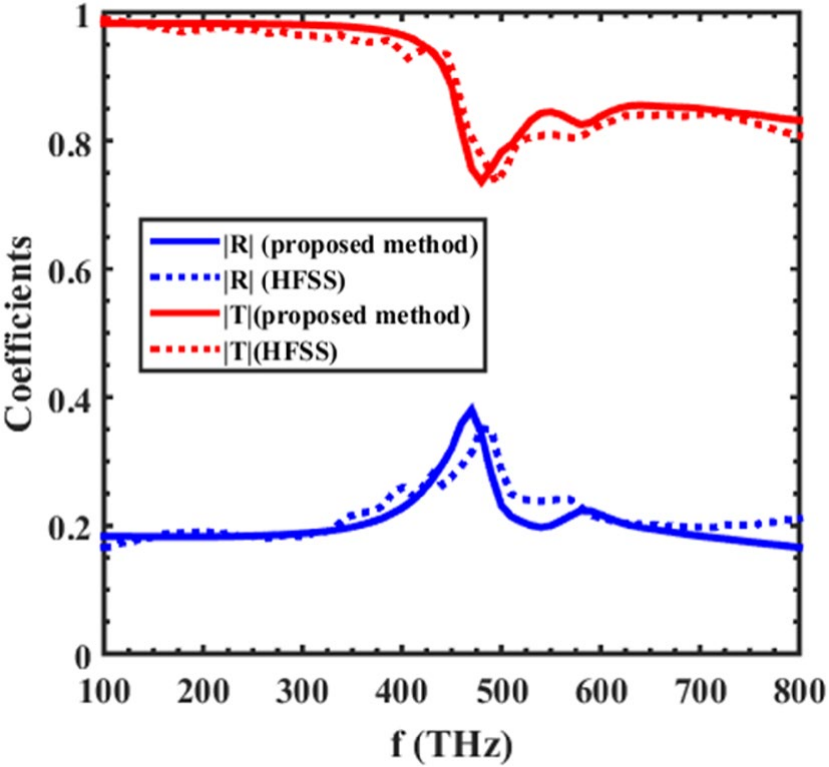
The amplitude of the reflection and transmission coefficients of the array presented in Fig. 6.

The next example deals with a supercell consisting of gold spherical and single-split cone particles located on an SiO2 substrate, where the schematics of this metasurface are shown in Fig. 6. In contrast to the previous example, a single-split cone particle has an electromagnetic polarizability due to its own bianisotropic features. This metasurface has been analyzed according to the procedure above, and the main components of the polarizabilities of the supercell are depicted in Fig. 7. The reflection and transmission coefficients of the substrated inhomogeneous metasurface have also been calculated using Eq. (3) mentioned in^18^, for a normal illumination. Figure 8 shows the results of our proposed approach, which have been compared to those obtained from a full-wave Ansys Inc. HFSS simulator, with the results showing good accordance.

As mentioned previously, our proposed approach is general, and can be applied to any arbitrary particle in free space or on a dielectric substrate; thus, these examples have only been presented to validate our proposed method. Moreover, it is worth mentioning that analyzing a metasurface by independently considering the effects of its individual nanoparticles, and the interactions between them, simplifies the array’s analysis. It is also useful for the synthesis of metasurfaces for different applications.

### An array of core–shell spherical particles as a wideband THz absorber

**Figure 9:**
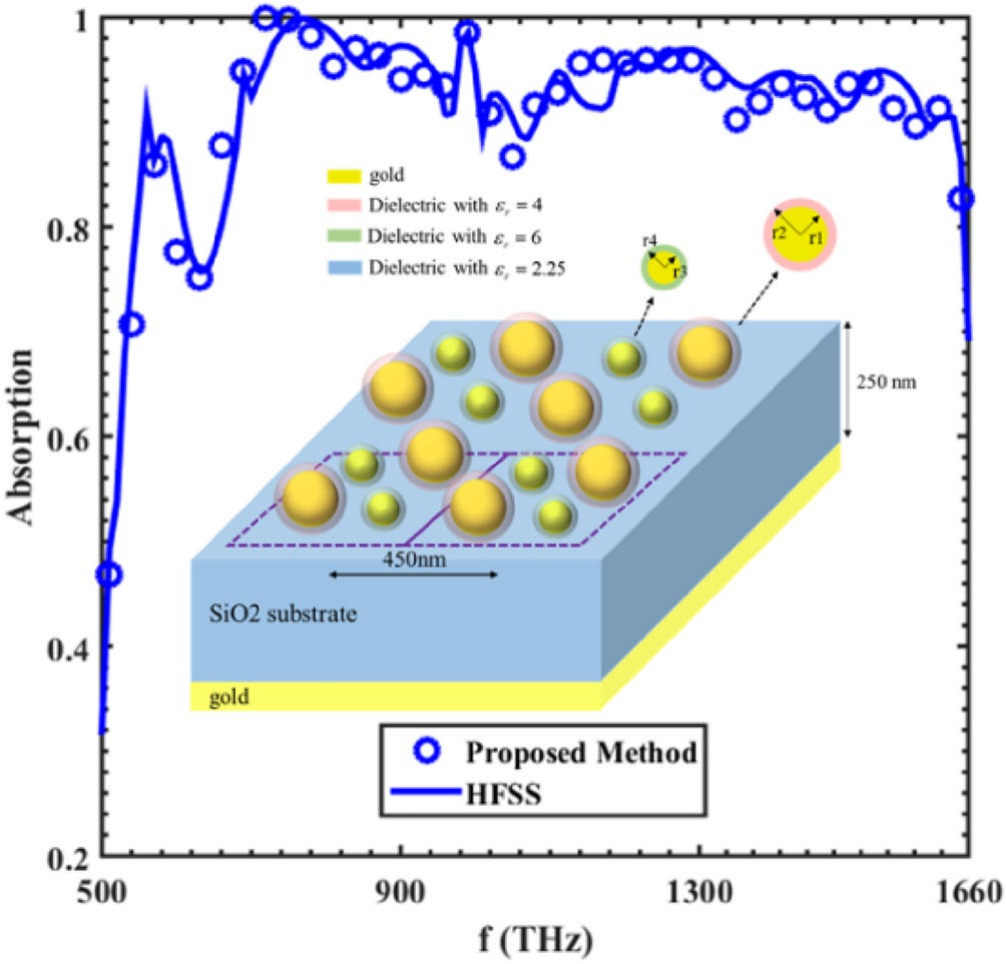
Absorption spectra for the proposed core–shell structure. See inset for structure.

The last example deals with a wideband THz absorber made of a periodic array of supercell located on top of a metallic ground plate, separated by a dielectric spacer. This supercell composed of core–shell particles as shown in Fig. 9. The metallic ground plate is used to suppress the transmission and increase the absorption.

To evaluate the accuracy and efficiency of the proposed ICM in analyzing this absorber with $$r_{1} = 90\begin{array}{*{20}c} {nm} \\ \end{array} ,\begin{array}{*{20}c} {r_{2} = 100} \\ \end{array} \begin{array}{*{20}c} {nm,\begin{array}{*{20}c} {r_{3} = 40\begin{array}{*{20}c} {nm,\begin{array}{*{20}c} {r_{4} = 50\begin{array}{*{20}c} {nm,} \\ \end{array} } \\ \end{array} } \\ \end{array} } \\ \end{array} } \\ \end{array}$$ first, the process summarized in the previous section is applied to calculate the reflection and transmission coefficients of the metasurface located on an infinite substrate. Then, by using the reflection and transmission coefficients calculated with our proposed method and multiple reflection method that presented in^21^, one can easily find the absorption of the structure. Figure 9 shows the absorption of the structure as a function of frequency using our proposed method and compared with HFSS result. As illustrated in the figure, the obtained results using ICM agree well with those of HFSS.

## Conclusion

In this paper, we extended the semi-analytical method for the efficient analysis of plane-wave scattering in the general case of inhomogeneous metasurfaces, in the presence of dielectric substrates, which could be considered as arrays of periodic supercells. Each supercell can be considered as a different particle with different shapes and materials. In our proposed method, by calculating the new interaction coefficients in the general form of an inhomogeneous metasurface, located at the interface between two dielectric media, one can now analyze planar homogeneous and inhomogeneous metasurfaces located in free space or on dielectric substrates, and illuminated by normal plane waves. The generality and validity of our proposed method are investigated using three examples that included both isotropic and bianisotropic particles with different materials. The results of our proposed method compared to those obtained by a commercial EM solver, and in each case, the two sets of results are in good agreement. Additionally, since in our proposed method, the interaction coefficients are calculated analytically, and have closed-form relations, the method is time-effective compared to both numerical methods and commercial software.

## Supplementary Information

Supplementary Information 1.

## Data Availability

The datasets generated and analyzed during the current study are available from the corresponding author on reasonable request.
