# Supplementary material for: Substrated inhomogeneous metasurfaces analysis using interaction constant method
Source: Sci Rep. 2023 Jan 28;13:1584. doi: 10.1038/s41598-023-28728-4 (PMC9884214; doi:10.1038/s41598-023-28728-4)
Supplement: Supplementary file 1 — Supplementary Information 1. [file 41598_2023_28728_MOESM1_ESM.docx]

Supplementary material: Substrated inhomogeneous metasurfaces analysis using interaction constant method

Maryam Hesari-Sherme ,^1,2^ Bijan Abbasi-Arand ,^1,2*^

^1^ Department of Electrical and Computer Engineering, Tarbiat Modares University, Tehran, 14115-194, Iran

2 Iran National Science Foundation (INSF), Tehran, Iran

*[abbasi@modares.ac.ir](mailto:abbasi@modares.ac.ir)

‎CALCULATIONG ELECTRIC AND MAGNETIC DIPOLE MOMENTS

‎In this Appendix‎, ‎we extend the far-field method presented in [1] to calculate the induced dipole moments for an arbitrary particle located on a substrate‎. ‎As the name of this method suggests‎, ‎it uses far scattered fields to calculate the moments; therefore‎, ‎it would be useful compared to other methods for calculating moments [1-3]‎.

‎To clarify the efficiency of this set-up‎, ‎compared to the set-ups mentioned in the literature‎, ‎it should be highlighted that‎, ‎as shown in Fig‎. S‎1‎, ‎we have considered the incident waves only from the upper environment‎. ‎Therefore‎, ‎if the substrate is a metal‎, ‎this setup can also be used‎. ‎Since for a metal substrate‎, ‎there is no field in the lower environment and it is also not possible to radiate waves from the second medium to the structure‎, ‎it is necessary for all the waves to be radiated to the structure from the upper environment‎, ‎and the sampling should be done from the upper environment‎.


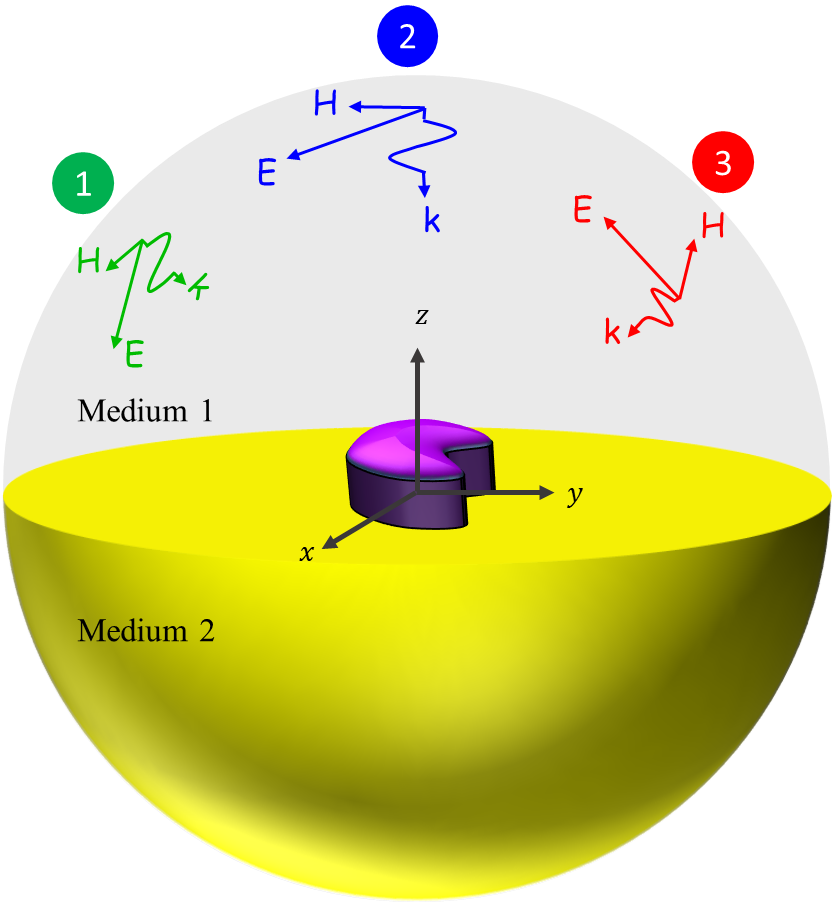


**Figure S1.** A polarizability tensor extraction setup, where an arbitrary particle is located at the interface between two different media, with permittivities $\mathcal{E}_{1}$ and $\mathcal{E}_{2}$.

‎Now‎, ‎let us consider the structure in Fig‎. S‎1‎. ‎Suppose a wave with (p) polarization in the *x‑z* plane is radiated to the particle under test (PUT)‎, ‎under oblique radiation‎, ‎and at an angle‎, $\theta_{i}$‎. ‎By radiating electromagnetic waves to the particle‎, ‎the electric and magnetic moments would be induced on the particle in its geometric center‎. ‎These moments would radiate in the presence of the substrate‎. ‎To compute these moments‎, ‎one could sample the scattered fields in certain directions‎. ‎To clarify this issue‎, ‎consider the scattered fields in the above environment‎, ‎which can be defined as follows‎:

| $\mathbf{E}^{up}=\mathbf{E}_{D}+\mathbf{E}_{R}$ | (S1) |
| --- | --- |

‎By using the Green's function of the scattered fields of an electric and magnetic dipoles as presented in [4]‎, ‎and considering the observation point is in the *x‑z* plane‎, ‎the scattered far-fields in the above medium can be written as follows‎:

|  | (S2) |
| --- | --- |

where $\gamma_{1}=\frac{k_{0}^{2}}{4\pi\varepsilon_{0}}$, $\gamma_{2}=e^{-2jk_{1}z_{0}}\gamma_{1}$, and $\theta$ is the observation point‎. ‎As can be seen in Eq‎. ‎(S2) that for the radiation of a plane-wave with an angle‎, $\theta_{i}$‎, ‎in order to calculate the induced moments‎, ‎it would be necessary to sample the fields at three separate observation points‎. ‎We consider these three observation points as $\theta_{o_{1}}$‎, $\theta_{o_{2}}$and $\theta_{o_{3}}$‎. ‎In order to find the unknown dipole moments‎, ‎Eq‎. ‎(S2) can be written in the following matrix form‎:

| $\begin{matrix} \left[\begin{matrix} E_{x}^{up}\left(\theta_{o_{1}}\right) \\ E_{x}^{up}\left(\theta_{o_{2}}\right) \\ E_{x}^{up}\left(\theta_{o_{3}}\right) \end{matrix}\right]=\left[\begin{matrix} X_{p}(o_{1}) & Z_{p}(o_{1}) & Y_{m}(o_{1}) \\ X_{p}(o_{2}) & Z_{p}(o_{2}) & Y_{m}(o_{2}) \\ X_{p}(o_{3}) & Z_{p}(o_{3}) & Y_{m}(o_{3}) \end{matrix}\right]\left[\begin{matrix} p_{x} \\ p_{z} \\ m_{y} \end{matrix}\right] \\ \left[\begin{matrix} E_{y}^{up}\left(\theta_{o_{1}}\right) \\ E_{y}^{up}\left(\theta_{o_{2}}\right) \\ E_{y}^{up}\left(\theta_{o_{3}}\right) \end{matrix}\right]=\left[\begin{matrix} Y_{p}(o_{1}) & X_{m}(o_{1}) & Z_{m}(o_{1}) \\ Y_{p}(o_{2}) & X_{m}(o_{2}) & Z_{m}(o_{2}) \\ Y_{p}(o_{3}) & X_{m}(o_{3}) & Z_{m}(o_{3}) \end{matrix}\right]\left[\begin{matrix} p_{y} \\ m_{x} \\ m_{z} \end{matrix}\right] \end{matrix}$ | (S3) |
| --- | --- |

Where

|  | (S4) |
| --- | --- |

‎Where *i*=1, 2, 3‎. ‎Now‎, ‎without losing the generality of the problem‎, ‎and for simplicity‎, ‎we assume that the observation points are in $\theta_{o_{1}}=-\theta_{o_{2}}=45^{\circ}$and $\theta_{o_{3}}=0^{\circ}$‎. ‎Solving the above equations gives the following expressions for the electric and magnetic dipole moments‎:

|  | (S5) |
| --- | --- |

‎This way‎, ‎the dipole moments can be calculated from the sampled scattered fields‎, ‎through Eq‎. ‎(S5)‎.

‎LOCAL EFFECTIOVE POLARIZABILITY TENSOR CALCULATION

‎To calculate the local effective polarizabilities of each particle in a supercell‎, ‎we have to calculate the relationships between the induced moments and the radiation fields; which are defined as follows‎:

| $\begin{matrix} \mathbf{p}_{i}=\bar{\bar{\hat{\alpha}}}_{i}^{ee}.\mathbf{E}^{inc}+\bar{\bar{\hat{\alpha}}}_{i}^{em}.\mathbf{H}^{inc}, \\ \mathbf{m}_{i}=\bar{\bar{\hat{\alpha}}}_{i}^{me}.\mathbf{E}^{inc}+\bar{\bar{\hat{\alpha}}}_{i}^{mm}.\mathbf{H}^{inc}, \end{matrix}$ | (S6) |
| --- | --- |

where the subscript, *i*, specifies the *i*th particle in the supercell. In the above equation, $\bar{\bar{\hat{\alpha}}}_{i}^{ee}$, $\bar{\bar{\hat{\alpha}}}_{i}^{em}$, $\bar{\bar{\hat{\alpha}}}_{i}^{me}$ and $\bar{\bar{\hat{\alpha}}}_{i}^{mm}$ are the electric‎, ‎magneto-electric‎, ‎electromagnetic and magnetic local effective polarizabilities of the *i*th supercell’s particle‎, ‎respectively‎. ‎As mentioned previously‎, ‎the local field for a particle located next to other particles is a superposition of the incident field and the interaction fields created by the other particles‎. ‎Therefore‎, ‎by replacing Eq‎. ‎(11) in Eq‎. ‎(1)‎, ‎for each particle in the supercell‎, ‎we have the following‎:

| $\left[\begin{matrix} \mathbf{p}_{i} \\ \mathbf{m}_{i} \end{matrix}\right]=\left[\begin{matrix} \bar{\bar{\alpha}}_{i}^{ee} & \bar{\bar{\alpha}}_{i}^{em} \\ \bar{\bar{\alpha}}_{i}^{me} & \bar{\bar{\alpha}}_{i}^{mm} \end{matrix}\right]\bullet\left[\begin{matrix} \mathbf{E}_{inc}+\sum_{\begin{matrix} j=1 \\ i\neq j \end{matrix}}^{N} \bar{\bar{\beta}}_{ji}^{ee}.\begin{matrix} \mathbf{p}_{j} \end{matrix}+\sum_{\begin{matrix} j=1 \\ i\neq j \end{matrix}}^{N} \bar{\bar{\beta}}_{ji}^{em}.\begin{matrix} \mathbf{m}_{j} \end{matrix} \\ \mathbf{H}_{inc}+\sum_{\begin{matrix} j=1 \\ i\neq j \end{matrix}}^{N} \bar{\bar{\beta}}_{ji}^{me}.\begin{matrix} \mathbf{p}_{j} \end{matrix}+\sum_{\begin{matrix} j=1 \\ i\neq j \end{matrix}}^{N} \bar{\bar{\beta}}_{ji}^{mm}.\begin{matrix} \mathbf{m}_{j} \end{matrix} \end{matrix}\right],$ | (S7) |
| --- | --- |

‎By solving Eq‎. ‎(S7) for all the particles in the supercell‎, ‎the local effective polarizabilities can be obtained using the following equation‎:

|  | (S8) |
| --- | --- |

where:

| $\left[\alpha^{t}\right]_{6N\times6N}= \left[\begin{matrix} I & 0 & \bar{\bar{\delta}}_{12}^{ee} & \bar{\bar{\delta}}_{12}^{em} & \cdots& \bar{\bar{\delta}}_{1N}^{ee} & \bar{\bar{\delta}}_{1N}^{em} \\ 0 & I & \bar{\bar{\delta}}_{12}^{me} & \bar{\bar{\delta}}_{12}^{mm} & \cdots& \bar{\bar{\delta}}_{1N}^{me} & \bar{\bar{\delta}}_{1N}^{me} \\ \bar{\bar{\delta}}_{21}^{ee} & \bar{\bar{\delta}}_{21}^{em} & I & 0 & \cdots& \bar{\bar{\delta}}_{2N}^{ee} & \bar{\bar{\delta}}_{2N}^{em} \\ \bar{\bar{\delta}}_{21}^{me} & \bar{\bar{\delta}}_{21}^{mm} & 0 & I & \cdots& \bar{\bar{\delta}}_{2N}^{me} & \bar{\bar{\delta}}_{2N}^{mm} \\ & \ddots& \ddots& & \ddots& & \\ \bar{\bar{\delta}}_{N1}^{ee} & \bar{\bar{\delta}}_{N1}^{em} & \bar{\bar{\delta}}_{N2}^{ee} & \bar{\bar{\delta}}_{N2}^{ee} & \cdots& I & 0 \\ \bar{\bar{\delta}}_{N1}^{me} & \bar{\bar{\delta}}_{N1}^{mm} & \bar{\bar{\delta}}_{N2}^{me} & \bar{\bar{\delta}}_{N2}^{me} & \cdots& 0 & I \end{matrix}\right]^{-1}.\left[\begin{matrix} \alpha_{1}^{ee} & \alpha_{1}^{em} & 0 & 0 & \cdots& 0 & 0 \\ \alpha_{1}^{me} & \alpha_{1}^{mm} & 0 & 0 & \cdots& 0 & 0 \\ 0 & 0 & \alpha_{2}^{ee} & \alpha_{2}^{em} & \cdots& 0 & 0 \\ 0 & 0 & \alpha_{2}^{me} & \alpha_{2}^{mm} & \cdots& 0 & 0 \\ & \ddots& \ddots& & \cdots& & \\ 0 & 0 & 0 & 0 & \cdots& \alpha_{N}^{ee} & \alpha_{N}^{em} \\ 0 & 0 & 0 & 0 & \cdots& \alpha_{N}^{me} & \alpha_{N}^{mm} \end{matrix}\right],$ | (S9) |
| --- | --- |

and we have:

| $\begin{matrix} \bar{\bar{\delta}}_{ij}^{ee}=-\left(\bar{\bar{\alpha}}_{i}^{ee}\left(\bar{\bar{\beta}}_{ij}^{ee}+\bar{\bar{\beta}}_{ij}^{'ee}\right)+\bar{\bar{\alpha}}_{i}^{em}\left(\bar{\bar{\beta}}_{ij}^{me}+\bar{\bar{\beta}}_{ij}^{'me}\right)\right), \\ \bar{\bar{\delta}}_{ij}^{em}=-\left(\bar{\bar{\alpha}}_{i}^{ee}\left(\bar{\bar{\beta}}_{ij}^{em}+\bar{\bar{\beta}}_{ij}^{'em}\right)+\bar{\bar{\alpha}}_{i}^{em}\left(\bar{\bar{\beta}}_{ij}^{mm}+\bar{\bar{\beta}}_{ij}^{'mm}\right)\right), \\ \bar{\bar{\delta}}_{ij}^{me}=-\left(\bar{\bar{\alpha}}_{i}^{me}\left(\bar{\bar{\beta}}_{ij}^{ee}+\bar{\bar{\beta}}_{ij}^{'ee}\right)+\bar{\bar{\alpha}}_{i}^{mm}\left(\bar{\bar{\beta}}_{ij}^{me}+\bar{\bar{\beta}}_{ij}^{'me}\right)\right), \\ \bar{\bar{\delta}}_{ij}^{mm}=-\left(\bar{\bar{\alpha}}_{i}^{me}\left(\bar{\bar{\beta}}_{ij}^{em}+\bar{\bar{\beta}}_{ij}^{'em}\right)+\bar{\bar{\alpha}}_{i}^{mm}\left(\bar{\bar{\beta}}_{ij}^{mm}+\bar{\bar{\beta}}_{ij}^{'mm}\right)\right). \end{matrix}$ | (S10) |
| --- | --- |

**References**

**1.** V. S. Asadchy, I. A. Faniayeu, Y. Radi, and S. A. Tretyakov, “Determining polarizability tensors for an arbitrary small electromagnetic scatterer, Photon,” *Nanostruct.: Fundam. Appl.*, **12**(4), 298304 (2014).

**2.** S. K. Patel, V. Sorathiya, T. K. Nguyen, and V. Dhasarathan, “Numerical investigation of tunable metasurface of graphene split-ring resonator for terahertz frequency with reflection controlling property,” *Physica E*, 118, p. 113910 (2020).

**3.** T. Niemi, A. O. Karilainen, and S. Tretyakov, Synthesis of polarization transformers, *IEEE Trans. Antennas Propag.*, **61**(6), pp. 31023111 (2013).

**4.** A.B. Evlyukhin, et al., "Multipole analysis of light scattering by arbitrary-shaped nanoparticles on a plane surface," *JOSA B*, **30**(10): p. 2589-2598 (2013).
